# Rotavirus Genotype Dynamics and the Emergence of G3P[8] in Thailand Following Nationwide Vaccine Implementation

**DOI:** 10.3390/ijms26189249

**Published:** 2025-09-22

**Authors:** Nutthawadee Jampanil, Kattareeya Kumthip, Thitapa Longum, Zhenfeng Xie, Arpaporn Yodmeeklin, Sirinart Sirilert, Nuthapong Ukarapol, Naphatrapee Sansaard, Channat Promping, Shoko Okitsu, Takeshi Kobayashi, Hiroshi Ushijima, Niwat Maneekarn, Pattara Khamrin

**Affiliations:** 1 Department of Microbiology, Faculty of Medicine, Chiang Mai University, Chiang Mai 50200, Thailand; 2 Emerging and Re-Emerging Diarrheal Viruses Cluster, Chiang Mai University, Chiang Mai 50200, Thailand; 3 Department of Obstetrics and Gynecology, Faculty of Medicine, Chiang Mai University, Chiang Mai 50200, Thailand; 4 Department of Pediatrics, Faculty of Medicine, Chiang Mai University, Chiang Mai 50200, Thailand; 5 Department of Medical Technology and Clinical Pathology, Sansai Hospital, Chiang Mai 50290, Thailand; 6 Medical Laboratory, Sanpatong Hospital, Chiang Mai 50290, Thailand; 7 Division of Microbiology, Department of Pathology and Microbiology, Nihon University School of Medicine, Tokyo 173-8610, Japan; 8 Department of Virology, Research Institute for Microbial Diseases, The University of Osaka, Osaka 565-0871, Japan

**Keywords:** rotavirus, epidemiology, gastroenteritis, diarrhea, Thailand

## Abstract

Rotavirus A is a leading cause of acute gastroenteritis in infants and young children under the age of five worldwide. The introduction of two live-attenuated oral vaccines, Rotarix and RotaTeq, has significantly decreased illness and death associated with rotavirus in countries where they are included in childhood immunization schedules. In Thailand, these two vaccines have been part of the national childhood immunization program since 2020. To monitor the changing patterns of rotavirus genotype distribution in the post-vaccination era, a molecular epidemiological study of rotavirus A was conducted in pediatric patients with acute diarrhea in Chiang Mai from 2020 to 2023, which was the period after the rotavirus vaccine was implemented in Thailand. A total of 1192 stool specimens collected from children with acute gastroenteritis were screened for rotavirus A by real-time PCR. The G- and P-genotypes were determined by using semi-nested PCR and nucleotide sequencing. A total of 60 out of 1192 (5.0%) samples were positive for rotavirus A. Among these, G3P[8] (55.0%) was identified as the most prevalent genotype, followed by G8P[8] (15.0%), G1P[8] (13.2%), G9P[8] (3.3%), G2P[4] (3.3%), G1P[6] (1.7%), G9P[4] (1.7%), and G8P[X] (1.7%). Additionally, the unusual rotavirus strains G3P[9] (1.7%), G3P[23] (1.7%), and G5P[23] (1.7%) were detected in this study. Phylogenetic analysis of the VP7 and VP4 genes revealed that the G3P[9] strain was closely related to those of feline rotaviruses, while the G3P[23] and G5P[23] strains showed high similarity to those of the porcine rotavirus strains detected previously in Thailand. This study demonstrated a significant decline in the prevalence of rotavirus A infection in pediatric patients in Chiang Mai, Thailand, during the post-vaccination period. The findings from this study contribute to a better understanding of rotavirus epidemiology in Thailand following the implementation of the rotavirus vaccines.

## 1. Introduction

Rotavirus (RV) is a leading viral pathogen that causes gastroenteritis in children under the age of five worldwide. A high RV infection rate was observed in children below two years of age [1,2]. Of nine RV species (A, B, C, D, F, G, H, I, and J), rotavirus A (RVA) is considered the most predominant causative agent of enteric diseases in humans [3,4]. RV is a non-enveloped virus with viral particles of 65–70 nm in diameter that is classified into the family *Sedoreoviridae* in the order *Reovirales* [3]. Its genomes consist of eleven segments of double-stranded RNA (dsRNA) enclosed within a triple-layered capsid. Each gene segment codes for a protein with a distinct function. The outer capsid proteins, VP4 and VP7, are responsible for virus attachment and cell entry. The middle capsid layer is formed by VP6, which is critical for determining the species, group, and subgroup of RV. The inner capsid layer consists of VP2, which encases the viral genome along with minor proteins VP1 (RNA-dependent RNA polymerase) and VP3 (capping enzyme) [5,6]. Except for gene segment eleven, all other genome segments encode either structural viral proteins (VP1–VP4, VP6, and VP7) or non-structural proteins (NSP1–NSP5/NSP6). [7,8]. RV is classified by using a dual system based on the outer capsid proteins VP7 (G glycoprotein) and VP4 (P protease-sensitive protein), which define the G- and P-genotypes, respectively [9]. To date, at least 42 G-genotypes and 58 P-genotypes have been detected in both humans and animals [10].

RV is highly infectious and spreads through the fecal–oral route, either by direct person-to-person contact or through contaminated surfaces, food, or water [11]. The symptoms typically begin within 48 h after infection, including a sudden fever and vomiting, followed by episodes of watery diarrhea. The infection may last three to eight days [12]. RVA infection leading to severe gastroenteritis has been reported worldwide with a prevalence of approximately 30–50%. The estimate of 128,500 annual deaths, with 258,173,300 episodes of diarrhea and 1,537,000 hospitalizations among children younger than five years of age worldwide, has been reported. The majority of deaths occurred in African, Oceanian, and South Asian countries during the past three decades [13]. Moreover, the RVA strains G1P[8], G2P[4], G3P[8], G4P[8], G8P[8], G9P[8], and G12P[8] are typically the most common genotypes that accounted for over 70% of all strains circulating in humans worldwide [14]. In Thailand, the infection rate of RVA among children hospitalized with diarrhea from 1977 to 2020 varied between 11.5% and 44.5%, and G1P[8], G2P[4], G3P[8], G8P[8], and G9P[8] were the most prevalent genotypes [15,16,17].

The introduction of live-attenuated oral RV vaccines is a significant public health achievement, leading to a remarkable decrease in the incidence of RV-related illnesses and deaths. Currently, there are two live-attenuated RV vaccines available globally, Rotarix (GlaxoSmithKline Biologicals, Rixensart, Belgium) and RotaTeq (Merck & Co. Inc., Rahway, NJ, USA), and the vaccine efficacies have been reported in the range of 85% to 98% in developing countries [18,19,20]. Additionally, India, China, and Vietnam have developed their own licensed RV vaccines, such as Rotasiil (Serum Institute of India Ltd., Pune, India), Rotavac (Bharat Biotec of Hyderabad, Telangana, India), LLR-85 (Lanzhou Institute of Biomedical Products, Lanzhou, China), and Rotavin-M1 (POLYVAC, Hanoi, Vietnam) [6,21]. Moreover, numerous RV vaccines are undergoing clinical trials worldwide [22]. Although the Rotarix and RotaTeq vaccines are part of national immunization programs implemented in more than 100 countries, RV-related mortality continues to rise in low-income countries [23,24]. In Thailand, a pilot study on the efficacy of RV vaccine (Rotarix) was conducted in two provinces, Sukhothai and Phetchabun, from 2012 to 2013 [25]. The findings indicated that Rotarix vaccine was highly effective in preventing hospitalized RV diarrhea and conferring herd protection to unvaccinated older children. Thereafter, two types of RV vaccines, Rotarix and RotaTeq, have been implemented in the National Immunization Program in Thailand since 2020 [26]. Several epidemiological studies have demonstrated the impact of RV vaccination on the distribution and diversity of RV genotypes [27,28]. Several studies have reported the change in RVA genotype distribution following vaccine introduction, suggesting possible vaccine-driven strain selection, particularly with the emergence of non-vaccine genotypes [24,29,30]. These findings indicate that the change in RVA strains may be impacted by RV vaccination. In Thailand, vaccine coverage has gradually increased since national implementation, which may influence genotype distribution. However, the role of vaccination in changing RVA genotype remains unclear in Thailand. In addition, evidence of zoonotic transmission of RVA has been reported in Thailand, suggesting that animal–human transmission may also affect the circulation of RVA genotype in this country. Therefore, continued monitoring of RVA genotype diversity in Thailand is essential. In the present study, we investigated molecular epidemiology and diversity of RVA genotypes following the introduction of RV vaccines in Thailand from 2020 to 2023.

## 2. Results

### 2.1. Detection Rate and Prevalence of RVA Infections

Among 1192 stool specimens collected from children under the age of five years who were admitted to hospitals with AGE, 5.0% were positive for RVA, as shown in Table 1. The prevalence of RVA infection decreased significantly from 6.2% in 2020 to 3.3% and 1.9% in 2021 and 2022, respectively, and prevalence rose to 8.2% in 2023. It should be noted that from 2021 to 2022, COVID-19 spread heavily, and restricted control measures were implemented in Thailand. To analyze the prevalence of RVA infection based on age and gender, the patients were stratified into five age groups: 0–1, >1–2, >2–3, >3–4, and >4–5 years old, as shown in Table 2. An analysis of RVA infections by age group showed that children aged > 2–3 years had the highest infection rate at 11.8%, followed by 8.2%, 4.5%, 2.0%, and 1.4% in the age groups of >3–4,  >1–2,  >0–1, and >4–5 years old, respectively. There was a statistically significant association between the RVA infection rate and age groups. The results indicated that RVA positivity rates vary significantly between particular age groups, specifically between children aged 0–1, >2–3, and >3–4 years old, with *p*-values of <0.001 and 0.004, respectively. Additionally, a significant difference was also observed between >1–2 years and >2–3 years, as well as between >2–3 years and >4–5 years, with *p*-values of 0.015 and 0.011, respectively. The other age groups did not show statistically significant differences. For the prevalence of RVA infection in different genders, the prevalence of RV infection was slightly higher in females (5.5%) than in males (4.9%). However, the difference was not statistically significant (Table 2).

### 2.2. Monthly Distribution of RVA Infection

**Figure 1:**
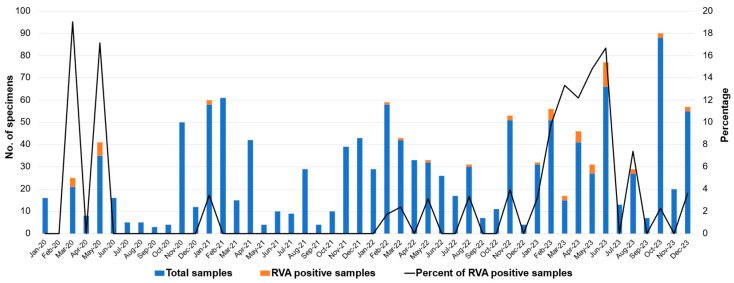
Monthly distribution of rotavirus A infection in pediatric patients with acute gastroenteritis from 2020 to 2023. No distinct seasonal pattern was observed from 2020 to 2022 (Chi-Square Test, *p* = 0.13). A clear seasonal peak emerged between February and June 2023 following the relaxation of COVID-19 control measures.

The seasonality of RVA infection was analyzed over a period of four years (2020 to 2023) as shown in Figure 1. In 2020, RVA-positive cases were detected only in March (19.0%) and May (17.1%), while in 2021, RVA infection was detected only in January (3.4%). No RVA-positive samples were found in the remaining months of these two years. Similarly, in 2022, RVA infection was detected only in February, March, May, August, and November with infection rates of 1.7%, 2.9%, 3.1%, 3.3%, and 3.9%, respectively. A chi-square test for uniformity showed no significant seasonal pattern from 2020 to 2022 (*p* = 0.13), which aligns with the disruption of typical viral transmission caused by COVID-19 control measures. In contrast, a distinct seasonal pattern re-emerged in 2023, RVA infection was detected almost all year round except in July, September, and November, with high infection rates in February (9.8%), March (13.3%), April (12.2%), May (14.8%), and June (16.7%). Overall, the COVID-19 pandemic affected the spread of RVA infection through the implementation of control measures, leading to a decline in RVA-positive cases and the absence of a distinct seasonal pattern from 2020 to 2022. However, a high frequency of RVA detection occurred in 2023, with the peak incidence spanning from February to June, which corresponded with the hot and dry months in Thailand.

### 2.3. Distribution of G- and P-Genotypes

The distribution of G- and P-genotypes of RVA strains in children with AGE are presented in Table 3. In 2020, G3P[8] (40.0%) was detected as the most prevalent genotype, followed by G2P[4] and G8P[8] (each at 20.0%), G1P[8] and G9P[8] (each at 10.0%). In 2021, G8P[8] (40.0%) was detected as the most predominant genotype, followed by G1P[8] (30.0%), G8P[X], G9P[4], and G9P[8] (each at 10.0%). Surprisingly, G3P[8], which was detected as the most prevalent genotype in 2020, was not detected in 2021. In 2022, G3P[8] (33.3%) re-emerged and became the most predominant genotype again, followed by G1P[6], G1P[8], G3P[23], and G8P[8] (each at 16.7%). In 2023, G3P[8] (79.5%) remained the most predominant genotype, as observed in 2020 and 2022, followed by, G1P[8] (8.8%), G8P[8] (5.9%), G3P[9], and G5P[23] (each at 2.9%). Overall, G3P[8] emerged as the most prevalent RVA strain (55.0%) among pediatric patients with AGE from 2020 to 2023 in Chiang Mai, Thailand. In addition, uncommon RVA strains, such as G1P[6], G3P[9], G3P[23], G5P[23], G9P[4], and G8P[X], were also detected in this study at a lower rate (each at 1.7%).

### 2.4. Phylogenetic Analysis of VP7 and VP4 Genes

**Figure 2:**
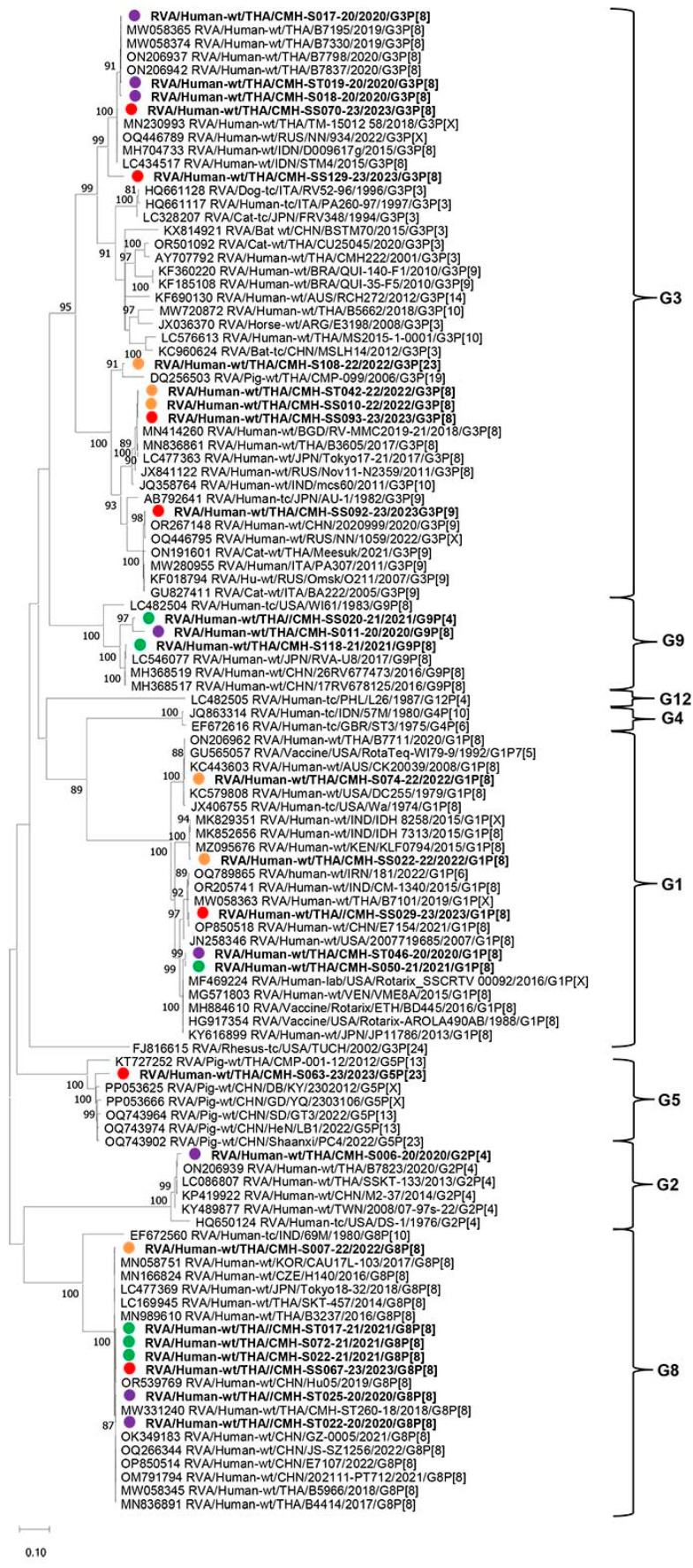
Phylogenetic analysis of rotavirus A based on partial nucleotide sequences of the VP7 gene (872 nt.). The tree was constructed using MEGA X software (version 10) via the Tamura 3-parameter+G+I as the best-fit evolutionary model. The selected rotavirus A strains detected in this study are displayed in boldface with purple (2020), green (2021), orange (2022), and red (2023) filled circles. Bootstrap values above 80% are displayed at individual nodes. The scale bars represent nucleotide substitutions per site.

To characterize the genetic backgrounds of the RVA strains detected in this study in comparison with the reference strains reported previously from various countries worldwide, phylogenetic analyses of their nucleotide sequences of VP7 and VP4 genes were analyzed. The phylogenetic tree of nucleotide sequences of the partial VP7 gene (872 nt) of the RVA strains detected in this study is shown in Figure 2. Among 27 representative sequences of VP7 included in this phylogenetic analysis, they represented six different G-genotypes, including G1, G2, G3, G5, G8, and G9. For the G1 genotype, five strains of G1 (CMH-S050-21, CMH-S074-22, CMH-SS022-22, CMH-SS029-23, and CMH-ST046-20) shared a close genetic relationship with human G1P[8] RVA reference strains and RV vaccine strains (Rotarix and RotaTeq) reported previously in Thailand, India, Iran, China, Japan, Kenya, and Venezuela, with high nucleotide sequence identities ranging from 91.2% to 99.4%. One strain of G2 (CMH-S006-20) clustered most closely with G2P[4] strain B7823 previously reported in Thailand with the nucleotide sequence identity of 98.8% and shared a close genetic relationship with human G2 RVA reference strains reported previously in Thailand, China, Taiwan, and the USA, with nucleotide sequence identities ranging from 92.0% to 98.3%. Four strains of G3 (CMH-S017-20, CMH-S018-20, CMH-ST019-20, and CMH-SS070-23) detected in this study clustered closely with G3 RVA strains previously reported in Thailand, Russia, and India, with nucleotide sequence identities ranging from 79.1% to 99.6%. One G3 strain, CMH-SS129-23, was also similar to the reference strains mentioned above with the nucleotide sequence identities ranging from 93.0% to 93.4% but located in a separate branch. Interestingly, one G3 human RVA strain (CMH-S108-22) displayed the highest nucleotide sequence identity (93.2%) with the G3P[23] strain (CMP-099) previously detected in a pig from Chiang Mai, Thailand, in 2006. The other three strains of G3 (CMH-ST042-22, CMH-SS010-22, and CMH-SS093-23) clustered closely with human G3 reference strains detected in Bangladesh, Thailand, Japan, India, and Russia, with nucleotide sequence identities ranging from 97.3% to 100%. Conversely, another G3 strain (CMH-SS092-23) formed a monophyletic branch with other G3P[9] genotype reference strains detected in China, Thailand, Italy, and Russia, with nucleotide sequence identities ranging from 98.4% to 99.8%. An uncommon human G5 RVA strain (CMH-S063-23) exhibited a close genetic relationship with four G5P[13] reference strains detected in pigs and wild boars from Thailand and China, with nucleotide sequence identities ranging from 94.2% to 96.0%. Seven representative RVA strains of G8 formed a cluster closely related to G8P[8] genotype reference strains reported from South Korea, the Czech Republic, Japan, Thailand, and China detected from 2014 to 2022, with nucleotide sequence identities ranging from 91.2% to 98.6%. In addition, three strains of G9 (CMH-SS020-21, CMH-S011-20, and CMH-S118-21) detected in this study located most closely in the same branch with human RVA G9P[8] strains reported previously from China and Japan, with nucleotide sequence identities ranging from 91.0% to 99.7%.

**Figure 3:**
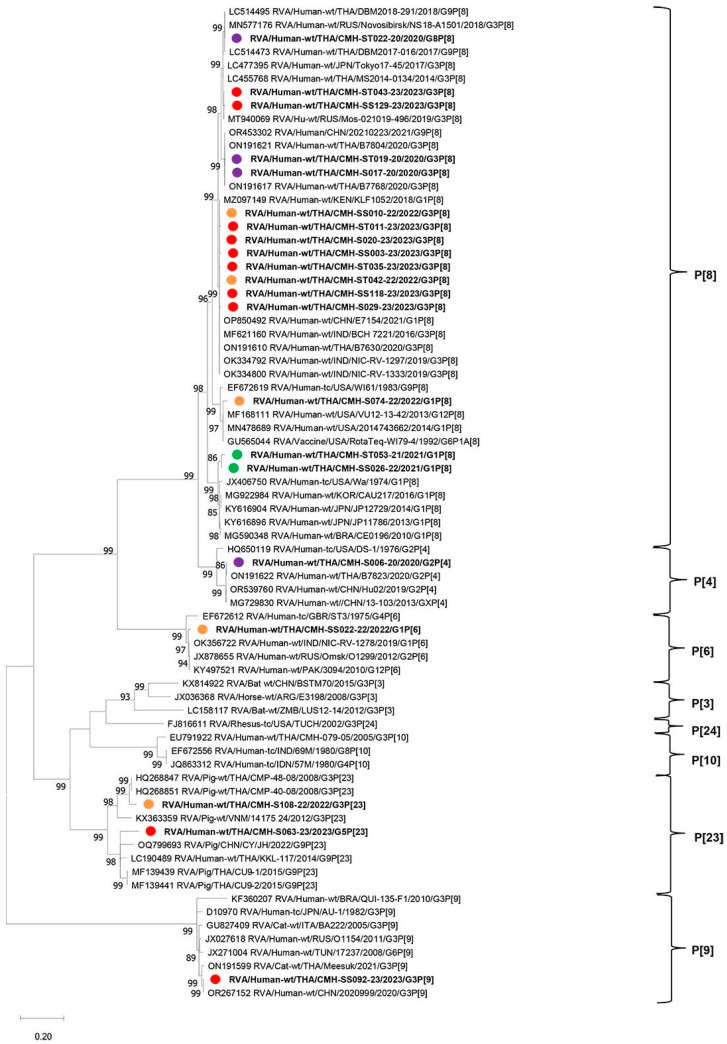
Phylogenetic analysis of rotavirus A based on partial nucleotide sequences of VP4 gene (820 nt.). The tree was constructed using MEGA X software via the Tamura 3-parameter+G+I as the best-fit evolutionary model. The selected rotavirus A strains detected in this study are displayed in boldface with purple (2020), green (2021), orange (2022), and red (2023) filled circles. Bootstrap values above 80% are displayed at individual nodes. The scale bars represent nucleotide substitutions per site.

The phylogenetic tree of partial VP4 nucleotide sequences (820 nt of the VP8* subunit) of the RVA strains detected in this study is shown in Figure 3. Among 21 VP4 representative sequences included in this phylogenetic tree analysis, they represented five different P-genotypes, including P[4], P[6], P[8], P[9], and P[23]. One strain of P[4] (CMH-S06-20) was closely related to human RVA reference strains of G2P[4] reported in Thailand and China, with very high nucleotide sequence identities ranging from 99.6% to 99.8%. Another P[6] strain (CMH-SS022-22) clustered closely together with G1P[6], G2P[6], and G12P[6] reference strains reported in India, Russia, and Pakistan, respectively, with nucleotide sequence identities ranging from 98.5% to 98.6%. For the P[8] genotype, one strain (CMH-ST22-20) exhibited the maximum nucleotide sequence identity (99.8%) with G3P[8] RVA reference strain NS18-A1501 reported previously in Russia. Two other P[8] strains (CMH-ST43-23 and CMH-SS129-23) were closely related to G3P[8] reference strains from Thailand, Japan, and Russia, with nucleotide sequence identities ranging from 99.0% to 99.5%. In addition, P[8] strains CMH-ST017-20 and CMH-ST019-20 showed the highest nucleotide sequence identity with those of Thai RVA G3P[8] strain B7768 at 99.8% and 99.6%, respectively. The other eight strains of P[8] (CMH-SS010-22, CMH-0SS003-23, CMH-SS118-23, CMH-ST042-22, CMH-ST011-23, CMH-ST035-23, CMH-S020-23, and CMH-S029-23) were closely related to the reference strain G1P[8] reported previously in Kenya and China and G3P[8] reported in India and Thailand, with nucleotide sequence identities ranging from 95.9% to 99.8%. Interestingly, the P[9] RVA strain CMH-SS092-23 detected in this study clustered closely together with the feline G3P[9] RVA strain Meesuk reported previously in Thailand in 2021. Additionally, two P[23] human RVA strains, CMH-S108-22 and CMH-S063-23, were similar to G3P[23] and G9P[23] RVA strains detected previously in pigs from Thailand, Vietnam, and China, with nucleotide sequence identities ranging from 81.5% to 96.0%.

## 3. Discussion

**Figure 4:**
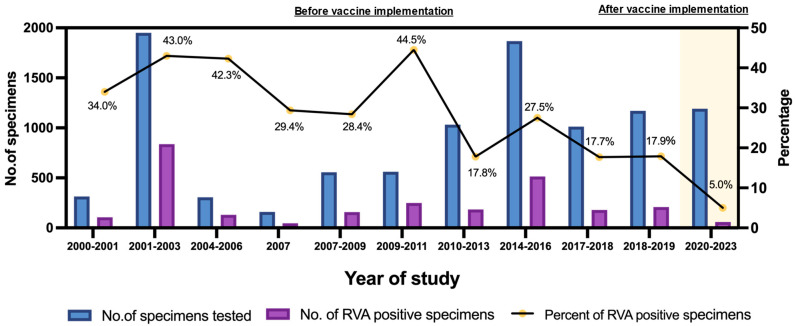
A comparison of the prevalence of rotavirus A infection in pediatric patients with acute gastroenteritis before and after rotavirus vaccine implementation in Thailand from 2000 to 2023. The data are based on previously published studies for the pre-vaccine era [15,16].

RVAs are the primary viral pathogens responsible for AGE in pediatric populations. The global burden of RV infection has decreased significantly in recent years in the regions where RV vaccination programs have been effectively implemented [31]. In Thailand, RotaTeq and Rotarix vaccines were first introduced as optional vaccines in 2005 and 2008, respectively [32]. Later, in 2020, RV vaccines were incorporated into the National Childhood Immunization Program. This study investigated the prevalence and genotype diversity of RVA in children with AGE from 2020 to 2023, which was the period after RV vaccines were implemented into the National Immunization Program in Thailand. This study compares the prevalence of RVA infection in children with AGE before and after RV vaccine use in Thailand, covering the years 2000 to 2023. The trend of the prevalence of RVA infection in pediatric patients with AGE in Thailand varies considerably from study to study and also depends on the study period, as shown in Figure 4. The 95% confidence intervals and error margins of RVA infection in pediatric patients with AGE in Thailand from 2000 to 2023 are summarized in Supplementary Figure S1 and Table S1. The prevalences during the period of 2000 to 2011 varied from 28.4% to 44.5%, and those from 2012 to 2019 ranged from 17.7% to 27.5% (the period before RV vaccine implementation), indicating that RV infection rates from 2000 to 2011 were relatively high, with the maximum rate being 44.5%, and the rates remarkably declined to 17.8%, 27.5%, 17.7%, and 17.9% from 2010 to 2013, from 2014 to 2016, from 2017 to 2018, and from 2018 to 2019, respectively [15,16]. After the implementation of RV vaccines in Thailand in 2020, the RV infection rate sharply dropped to 5% (average RV infection rate) from 2020 to 2023. This finding is most likely attributable to the effectiveness of RV vaccine implementation in Thailand. The widespread use of RV vaccines has been shown to significantly decrease the incidence of RV infections and provide indirect protection for unvaccinated people in the community [33]. The reduction in RVA infections in our study aligns with increasing national vaccine coverage during the study period. According to WHO and UNICEF estimates, national RV vaccine coverage in Thailand rose from about 30 to 55% in 2020 to over 80% in 2022 and reached 85 to 90% in 2023 [26,34]. Therefore, the decline in RVA prevalence rates may reflect the protective impact of RV vaccination in the pediatric population in Thailand. Moreover, the infection control measures implemented during the COVID-19 pandemic also likely contributed to the decline in RVA infection [35,36].

By the age of five, almost all children around the world experience at least one RV infection regardless of their geographical location or socioeconomic status [37]. Maternal antibodies are transferred from mother to infant and are effective in protecting neonates and children against most infectious diseases. However, maternal antibodies decline gradually over a period of 6 to 12 months [38]. Analysis of RVA infection in children with different age groups (Table 2) revealed that the RVA infection rate was 2.0% at the age of 0–1 years old and then increased remarkably to 4.5%, 11.8%, and 8.2% at the ages of >1–2, >2–3, and >3–4 years old, respectively. The data imply that the low infection rate in children aged 0–1 years might be due to protection by maternal antibodies passively transferred to the newborn via breastfeeding, and these maternal antibodies decline after 1 year of age. Consequently, the infection rate increased in children at older ages. The highest prevalence was detected in the >2–3-year age group (11.8%). Previous studies also reported that RV vaccine effectiveness decreases in the second year of life, especially in low- and middle-income countries [36,39]. Other factors, such as poor hygiene or the activities of older children, may increase the risk of RV infection [11].

This study was conducted during the period of the COVID-19 pandemic from 2020 to 2022 and extended to 2023 when the restricted control measures for COVID-19 were relaxed. The implementation of restricted control measures during the COVID-19 pandemic from 2020 to 2022 had a serious impact on the seasonal pattern of RVA infection. It has been demonstrated that COVID-19 control measures also had an impact on the incidence of other infectious diseases, including gastroenteritis [40,41,42]. For the seasonal analysis of RVA infection, statistical analysis indicated no significant variation between 2020 and 2022 (*p* > 0.05) (Figure 1). RVA infection was detected only in March and May in 2020, only in January in 2021, and only in February, March, May, August, and November in 2022. The seasonal pattern of RVA infection could not be drawn from this study period. However, after the relaxing of COVID-19 control measures in 2023, the seasonal pattern of RVA infection was observed with peak incidence spanning from February to June, which is during the hot and dry season in Thailand, and this pattern is slightly different from other studies showing that RVA infections in the pre-vaccine era usually peak during the cool and dry months (November to March) [15]. The unclear seasonality of RVA prevalence observed in this study may be explained by the relatively small number of RV-positive cases detected in each year (10 cases in 2020 and 2021, 6 cases in 2022, and 34 cases in 2023). Information on the numbers of specimens collected from AGE cases and the RVA-positive rates in each year is presented in Table 1. Globally, interventions during the period of the COVID-19 pandemic have been shown to reduce the transmission of not only SARS-CoV-2 but also other viral pathogens [43].

**Figure 5:**
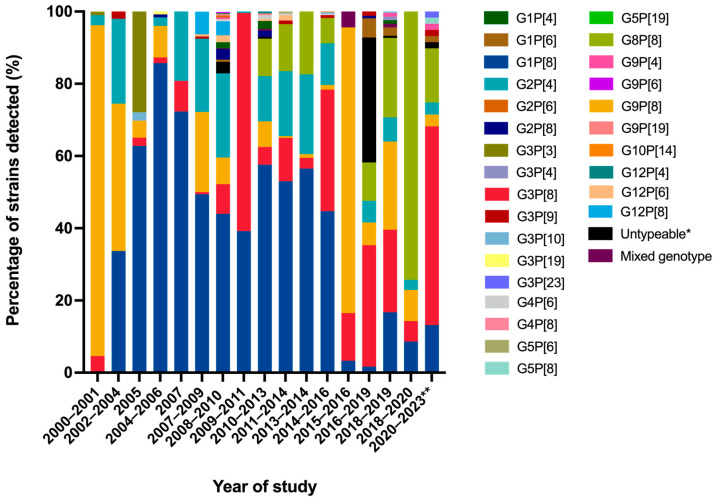
Distribution of G- and P-genotypes of rotavirus A strains circulating in pediatric patients with acute gastroenteritis before and after rotavirus vaccine implementation in Thailand from 2000 to 2023. * PCR products could not be amplified. ** G- and P-genotype distribution of rotavirus A detected after rotavirus vaccine implementation. Note: The data from 2000 to 2019, the period before rotavirus vaccine implementation in Thailand, are published in [15,16].

The distribution of G- and P-genotypes of RVA strains circulating in pediatric patients with AGE in Thailand has undergone significant changes over time (Figure 5). During the pre-vaccination era (2000 to 2019), G1P[8], G2P[4], and G9P[8] were identified as the most commonly detected genotypes. Among these, G1P[8] was the dominant RVA genotype, and it began to decline sharply to a very low detection rate from 2015 to 2019 [44,45]. Similarly, G2P[4] also showed a significant decline from 2016 to 2019, while G3P[8], G8P[8], and G9P[8] became more predominant during this period. Notably, G8P[8] emerged as the most prevalent genotype from 2018 to 2020, before RV vaccine implementation [46]. Although both Rotarix and RotaTeq vaccines are designed to protect against a broad range of human RVA strains, including G3P[8], following the implementation of RV vaccines into the National Immunization Program in Thailand in 2020, we observed an increase in the G3P[8] genotype, followed by G8P[8]. This shift in the prevalence of RVA genotype has been reported in many other regions as well. For example, G3P[8] emerged as the predominant genotype after RV vaccine introduction in Australia [28], Kenya [47], and Mozambique [48]. Similarly, the RVA genotype G8P[8] became predominant in the post-vaccine implementation period in Korea [49]. The rise in G3P[8] in recent years could be attributable to several factors. When a specific RV genotype circulates within a population over a period of time, herd immunity may potentially reduce the prevalence of common genotypes, such as G1P[8]. In addition, it is possible that RV vaccines may somehow influence the distribution of RV genotypes, leading to the emergence of some previously less common strains. This phenomenon creates an ecological niche that facilitates the emergence and spread of other genotypes, including the G3P[8] genotype [50]. The G3P[8] genotype may possess distinct antigenic properties that enable it to infect individuals who had partial immunity, therefore increasing its prevalence in the population [51,52]. Recently, novel equine-like human G3P[8] strains have emerged and spread across several countries. These strains are considered to be originated from a reassortment event involving equine RV strains and became the predominant genotype in many countries [53,54]. For example, in Brazil, G3P[8] strains detected between 2017 and 2020 were identified as equine-like G3P[8] RVs [29]. In Italy, a marked reduction in G1P[8] was observed, with the emergence of the equine-like G3P[8] strain in 2018 to 2019 after the introduction of Rotarix [24]. In Venezuela, equine-like G3P[8] strains were also detected in children with diarrhea between March 2023 and April 2024 [30]. Similarly, our analysis confirmed that the G3P[8] strains detected in this study were closely related to equine-like G3P[8] reference strains. These findings suggest that the equine-like G3P[8] strain emerged globally following RV vaccine introduction.

The segmented nature of the RVA genome facilitates genetic reassortment during co-infection, which can lead to the emergence of uncommon or novel RVA strains. Several studies reveal a rising number of uncommon RVA strains that appear to originate from interspecies transmission of RVA between humans and other animal host species such as dogs, cats, pigs, and horses [55]. In Thailand, uncommon RVA genotypes have occasionally been detected in children in Chiang Mai and other regions, indicating the circulation of animal-like strains within the human population [15,16]. Our study detected unusual RVA strains, including G3P[9], G3P[23], and G5P[23] genotypes. Based on the VP7 and VP4 gene analyses, the feline-like human RVA strain G3P[9] detected in this study was closely related to the G3P[9] strain reported previously in a 2-year-old indoor-housed female Siamese cat with bloody mucoid diarrhea in Bangkok, Thailand [56]. The G3P[9] strain is known to be one of the prevalent genotypes in feline RVs and has occasionally been detected in children in Thailand [57]. Therefore, our results indicate a potential cross-species transmission of RVA among humans and cats. In addition, the G3P[23] and G5P[23] strains detected in this study exhibit close genetic similarity to porcine RVA strains reported previously in Thailand, Vietnam, and China. In Northern Thailand, a high concentration of smallholder pig farms is located near residential communities. In addition, a recent study on diarrheic piglets in Northern Thailand reported that G3P[23] and G5P[23] were the predominant porcine RVA genotypes [58]. These may facilitate the transmission of viruses between animals and humans. Thus far, there is currently no clear evidence that these viral strains escape vaccine-induced immunity or cause more severe disease. Conducting pathogenesis experiments will provide a more comprehensive understanding of their potential health risks.

After the implementation of RV vaccines into the National Immunization Program in Thailand in 2020, the RVA-related hospitalization and diarrhea-related mortality rates in children decreased significantly. However, this study has some limitations. First, there were no records of individual vaccination status or clinical manifestations for the enrolled cases. Second, the NSP3 assay used for RVA detection cannot differentiate between vaccine-derived and wild-type RV strains. Third, only the VP7 and VP4 genes of RVA strains, particularly G3P[8], were analyzed. Therefore, performing a complete genome analysis would help clarify the evolutionary patterns of these RVA strains.

## 4. Materials and Methods

### 4.1. Study Design and Settings

A total of 1192 stool specimens were consecutively collected from pediatric patients under five years of age who were suffering from AGE and admitted in five major hospitals located in Chiang Mai, Thailand. The participating hospitals comprise one tertiary university hospital, two urban private hospitals, and two district hospitals. The specimen collection period was from January 2020 to December 2023. The inclusion criteria for AGE are defined as defecation of three or more loose or watery stools and an increase in the number of bowel movements in a 24 h period [59].

### 4.2. Viral RNA Extraction and RVA Detection by RT-qPCR

All viral RNA was extracted from 140 μL of 10% (*w*/*v*) stool suspension prepared in 1X phosphate-buffered saline (PBS, pH 7.4) using the QIAamp Viral RNA Mini Kit (Qiagen, Hilden, Germany) according to the manufacturer’s protocol. The extracted viral RNA was denatured by mixing with a 50% dimethyl sulfoxide (DMSO) solution and heating at 95 °C for 5 min and then cooled on ice immediately. The viral RNA was subsequently reverse-transcribed into complementary DNA (cDNA) using the RevertAid First Strand cDNA Synthesis kit with random primers (Thermo Scientific, Waltham, MA, USA) following the protocol provided by the manufacturer. Quantitative real-time PCR (qPCR) was used to screen the cDNA samples for RVA. For the specific detection of RVA, the conserved region within the NSP3 gene was amplified using the following oligonucleotide primers: forward primer JVKF (5′-CAGTGGTTGATGCTCAAGATGGA-3′), reverse primer JVKR (5′-TCATTGTAATCATATTGAATACCCA-3′), and probe JVKP (FAM-ACAACTGCAGCTTCAAAAGAAGWGT-MGB) [60]. The NSP3 gene was selected as the detection target because it is highly conserved among diverse RVA genotypes and provides high sensitivity and specificity [61,62]. It should be noted that this assay cannot distinguish between vaccine and wild-type strains. An amount of 20 μL of the qPCR reaction mixture was prepared from 10 μL of 2× THUNDERBIRD^®^ Probe qPCR Master Mix (TOYOBO, Tokyo, Japan), 0.8 μL (10 nM) each of the forward primer (JVKF) and reverse primer (JVKR), 0.4 μL of the JVKP probe, 6.0 μL of nuclease-free water, and 2.0 μL of cDNA template. The experiment was carried out on a CFX Opus Real-Time PCR System (Bio-Rad, Hercules, CA, USA). The thermocycling conditions included initial denaturation at 95 °C for 1 min, followed by 40 cycles at 95 °C for 15 s and 56 °C for 1 min. The results were analyzed using the CFX Maestro Software (version 4.1). The baseline was automatically determined, and the threshold was manually adjusted during the exponential phase of the amplification process.

### 4.3. RVA G- and P-Genotyping, Nucleotide Sequencing, and Phylogenetic Analysis

To determine the G- and P-genotypes of RVA, the semi-nested polymerase chain reaction (PCR) was performed on all RVA-positive samples. For G-genotyping, a partial VP7 gene of RVA was initially amplified using the sBeg9 forward primer paired with the End9(s) reverse primer [63,64]. For the semi-nested amplification of the VP7, a multiplex PCR for G-genotyping was subsequently performed using the sets of genotype-specific primers for G1-G4, G8, and G9 [65,66]. Similarly, for P-genotyping, the amplification of a partial VP4 gene was performed using the Con3 forward and Con2 reverse primers [65]. Then, semi-nested PCR for P-genotyping by multiplex PCR was performed using genotype-specific primers consisting of the forward primers and the reverse primers for determining the P[4], P[6], P[8], and P[19] genotypes [45,67]. The PCR mixture of 25.0 μL contained 5.0 μL of 5X Green GoTaq^®^ Reaction Buffer (Promega, Madison, WI, USA), 2.0 μL of 2.5 mM dNTP mix (Roche, Indianapolis, IN, USA), 2.0 μL of 20 μM specific primer pair, 0.1 μL of GoTaq^®^ DNA polymerase (Promega, USA), 1.0 μL of cDNA template, and 14.9 μL of RNase-free water. PCR amplification was carried out with initial denaturation at 94 °C for 3 min, followed by 35 cycles at 94 °C for 1 min, 48 °C for 1 min, 72 °C for 1 min, and a final extension at 72 °C for 10 min. Then, the PCR amplicons were analyzed on a 1.5% agarose gel.

The RVA-positive PCR products were further characterized by nucleotide sequencing and phylogenetic analysis. The PCR products derived from VP7 and VP4 genes were purified following the manufacturer’s procedure using a Gel/PCR DNA Fragments Extraction Kit (Geneaid, Xinbei, Taiwan). The purified PCR products were subjected to fluorescent dye terminator sequencing at First BASE Laboratories Sdn Bhd (Bangsar South City, Malaysia). The obtained nucleotide sequences of both VP7 and VP4 RVAs were analyzed using the Basic Local Alignment Search Tool (BLAST (BLAST+ 2.17.0)) available at http://blast.ncbi.nlm.nih.gov/Blast.cgi (accessed on 17 September 2025) and compared with those of the GenBank reference strains. The sets of representative nucleotide sequences were used to construct the phylogenetic trees for VP7 and VP4 genes using the maximum likelihood method in Molecular Evolutionary Genetics Analysis version 10 (MEGA X) software. The best-fit evolutionary models were determined based on the Bayesian information score (BIC) [68]. The model used in this study was Tamura 3-parameter+G+I (T92+G+I) for VP7 and VP4 sequences. To evaluate the robustness of the branching patterns, bootstrap analysis with 1000 replicates was performed. BioEdit software version 7.2 was used to analyze the nucleotide sequence identity.

### 4.4. Statistical Analysis

IBM SPSS version 29 software was used for statistical analyses. The significant differences in RVA infections across different ages and genders were assessed using the Chi-squared and Fisher’s exact tests as appropriate. For pairwise age group comparisons, Bonferroni correction was applied to adjust for multiple testing. To evaluate seasonal variation from 2020 to 2022, a chi-square goodness-of-fit test was performed. A *p*-value equal to or less than 0.05 (≤ 0.05) was considered statistically significant.

## Figures and Tables

**Table 1:** Prevalence of rotavirus A infection in pediatric patients with acute gastroenteritis from 2020 to 2023.

Year	Number of Specimen Tested	Number of RVA-Positive Specimens (%)
2020	161	10 (6.2%)
2021	303	10 (3.3%)
2022	313	6 (1.9%)
2023	415	34 (8.2%)
Total	1192	60 (5.0%)

**Table 2:** Prevalence of rotavirus A infection in different genders and age groups of pediatric patients with acute gastroenteritis.

Variable		Number of Cases	Rotavirus A Positive (%)	Rotavirus A Negative (%)
**Gender** ^a^	Male, *n* (%)	678 (56.9)	33 (4.9)	641 (94.5)
	Female, *n* (%)	490 (41.1)	27 (5.5)	467 (95.3)
	Total	1168 ^c^	60	1108 ^c^
**Age group (year)** ^b^	0–1	457	9 (2.0)	448 (98.0)
	>1–2	243	11 (4.5)	232 (95.5)
	>2–3	119	14 (11.8)	105 (88.2)
	>3–4	97	8 (8.2)	89 (91.8)
	>4–5	72	1 (1.4)	71 (98.6)
	Total	988 ^d^	43 ^d^	945 ^d^

^a^ Chi-Square test. ^b^ Fisher’s exact test. ^c^ Gender information of 24 cases was not recorded. ^d^ Age information of 204 cases was not recorded. Of these, 17 were positive for RVA. *p*-value of 0–1 vs. >2–3 ≤ 0.001 *; 0–1 vs.  >3–4  =  0.004 *; >1–2 vs.  >2–3  =  0.015 *; >2–3 vs. >4–5  =  0.011 *. * *p*-value equal to or less than 0.05 (≤ 0.05) is considered statistically significant.

**Table 3:** Distribution of G- and P-genotypes of human rotavirus A circulating in pediatric patients with acute gastroenteritis from 2020 to 2023.

Rotavirus A Genotype	Number of Rotavirus A Strains (%)				
	2020 (*n* = 161)	2021 (*n* = 303)	2022 (*n* = 313)	2023 (*n* = 415)	Total (*n* = 1192)
**G1P[6]**	0 (0.0)	0 (0.0)	1 (16.7)	0 (0.0)	1 (1.7)
**G1P[8]**	1 (10.0)	3 (30.0)	1 (16.7)	3 (8.8)	8 (13.3)
**G2P[4]**	2 (20.0)	0 (0.0)	0 (0.0)	0 (0.0)	2 (3.3)
**G3P[8]**	4 (40.0)	0 (0.0)	2 (33.3)	27 (79.5)	33 (55.0)
**G3P[9]**	0 (0.0)	0 (0.0)	0 (0.0)	1 (2.9)	1 (1.7)
**G3P[23]**	0 (0.0)	0 (0.0)	1 (16.7)	0 (0.0)	1 (1.7)
**G5P[23]**	0 (0.0)	0 (0.0)	0 (0.0)	1 (2.9)	1 (1.7)
**G8P[8]**	2 (20.0)	4 (40.0)	1 (16.7)	2 (5.9)	9 (15.0)
**G8P[X]**	0 (0.0)	1 (10.0)	0 (0.0)	0 (0.0)	1 (1.7)
**G9P[4]**	0 (0.0)	1 (10.0)	0 (0.0)	0 (0.0)	1 (1.7)
**G9P[8]**	1 (10.0)	1 (10.0)	0 (0.0)	0 (0.0)	2 (3.3)
**Total**	10 (16.7)	10 (16.7)	6 (10.0)	34 (56.6)	60 (100) *

* Percentages may not add up to 100.0 due to rounding.

## Data Availability

The data that support the findings of this study are openly available in the National Center for Biotechnology Information (NCBI) at https://www.ncbi.nlm.nih.gov/nucleotide/ accessed on 17 September 2025, accession numbers PV167218 to PV167265. These include 27 VP7 (G-genotypes) and 21 VP4 (P-genotypes) sequences.

## Supplementary Materials

The following supporting information can be downloaded at: https://www.mdpi.com/article/10.3390/ijms26189249/s1.

## Abbreviations

The following abbreviations are used in this manuscript:

AGE	Acute gastroenteritis
COVID-19	Coronavirus disease 2019
DNA	Deoxyribonucleic acid
nm	Nanometer
NSP	Nonstructural protein
PCR	Polymerase chain reaction
pH	Potential of hydrogen ion
RT	Reverse transcription
RT-qPCR	Reverse transcription quantitative polymerase chain reaction
RNA	Ribonucleic acid
Rnase	Ribonuclease
RV	Rotavirus
RVs	Rotaviruses
RVA	Rotavirus A
SARS-CoV-2	Severe acute respiratory syndrome coronavirus 2
VP	Viral protein
